# Molecular Profiling of Single Sca-1^+^/CD34^+,−^ Cells—The Putative Murine Lung Stem Cells

**DOI:** 10.1371/journal.pone.0083917

**Published:** 2013-12-31

**Authors:** Markus Hittinger, Zbigniew T. Czyz, Yves Huesemann, Matthias Maneck, Catherine Botteron, Stephanie Kaeufl, Christoph A. Klein, Bernhard Polzer

**Affiliations:** 1 Chair for Experimental Medicine and Therapy Research, University of Regensburg, Regensburg, Germany; 2 Institute of Functional Genomics, University of Regensburg, Regensburg, Germany; 3 Project Group “Personalized Tumor Therapy”, Fraunhofer Institute for Experimental Medicine and Toxicology, Regensburg, Germany; University of Central Florida, United States of America

## Abstract

Murine bronchioalveolar stem cells play a key role in pulmonary epithelial maintenance and repair but their molecular profile is poorly described so far. In this study, we used antibodies directed against Sca-1 and CD34, two markers originally ascribed to pulmonary cells harboring regenerative potential, to isolate single putative stem cells from murine lung tissue. The mean detection rate of positive cells was 8 per 10^6^ lung cells. We then isolated and globally amplified the mRNA of positive cells to analyze gene expression in single cells. The resulting amplicons were then used for molecular profiling by transcript specific polymerase chain reaction (PCR) and global gene expression analysis using microarrays. Single marker-positive cells displayed a striking heterogeneity for the expression of epithelial and mesenchymal transcripts on the single cell level. Nevertheless, they could be subdivided into two cell populations: *Sca-1^+^/CD34* ^−^ and *Sca-1^+^/CD34^+^* cells. In these subpopulations, transcripts of the epithelial marker Epcam (CD326) were exclusively detected in *Sca-1^+^/CD34* ^−^ cells (p = 0.03), whereas mRNA of the mesenchymal marker Pdgfrα (CD140a) was detected in both subpopulations and more frequently in *Sca-1^+^/CD34^+^* cells (p = 0.04). FACS analysis confirmed the existence of a Pdgfrα positive subpopulation within Epcam^+^/Sca-1^+^/CD34^−^ epithelial cells. Gene expression analysis by microarray hybridization identified transcripts differentially expressed between the two cell types as well as between epithelial reference cells and *Sca-1^+^/CD34^+^* single cells, and selected transcripts were validated by quantitative PCR. Our results suggest a more mesenchymal commitment of *Sca-1^+^/CD34^+^* cells and a more epithelial commitment of *Sca-1^+^/CD34* ^−^ cells. In summary, the study shows that single cell analysis enables the identification of novel molecular markers in yet poorly characterized populations of rare cells. Our results could further improve our understanding of Sca-1^+^/CD34^+,−^ cells in the biology of the murine lung.

## Introduction

The murine lung contains at least 40 morphologically distinct cell types of mesodermal or endodermal origin [1]. It can be subdivided anatomically in three different regions: large airways, bronchioles, and alveoli. Each region is composed of different cell types whose homeostasis is guaranteed by regionally specific progenitor cells [2], [3]. Importantly, Sca-1^+^ cells have been ascribed decisive functions regarding the maintenance and repair of the airways [2]. In injury models, Sca-1^+^ cells demonstrated resistance to damage and clonal expansion leading to a restoration of previously experimentally depleted epithelial structures [2]. During the last years, considerable efforts have been undertaken to further characterize a specific subgroup of Sca-1^+^ cells. These cells were named bronchioalveolar stem cells (BASCs) based on their location at the bronchioalveolar duct junction [4] where they proved to play a key role in distal airway repair [4]–[7].

However, a more detailed characterization of this subpopulation in terms of its protein expression remained challenging. Kim and colleagues [4] identified BASCs as positive for stem cell antigen 1 (Sca-1), surfactant protein C (Sftpc), clara cell secretory protein (Ccsp), and cluster of differentiation 34 (CD34) as well as negative for the platelet endothelial cell adhesion molecule (CD31, a marker of endothelial cells), and protein tyrosine phosphatase, receptor type C (CD45, a marker of hematopoietic cells). More recent studies, however, confirmed Sca-1 and Sftpc but not CD34 as potential marker proteins of BASCs [8]–[10]. In contrast to the original publication [4], Sca-1^+^/CD34^+^/CD45^−/^CD31^−^ cells showed no enhanced proliferation rate after lung injury as it could be demonstrated with Sca-1^+^/CD34^−/^CD45^−/^CD31^−^ cells [10]. Furthermore, Sca-1^+^/CD34^+^/CD45^−/^CD31^−^ cells co-expressed mesenchymal markers thymus cell antigen 1 (CD90) and platelet derived growth factor receptor α (Pdgfrα, CD140a) questioning their epithelial origin [8]. The epithelial cell adhesion molecule (Epcam, CD326), α6-Integrin (Itga), ß4-Integrin and cluster of differentiation 24 (CD24) were newly introduced as BASC-specific markers [2], [9], [10].

Considering the heterogeneity of Sca-1^+^ murine lung cells, as it could be demonstrated for the group of BASCs, the isolation and subsequent gene expression analysis of single cells may provide an interesting tool for the identification of novel molecular markers in poorly described rare cells [11], [12]. Eventually, this approach could allow better distinction between the different subtypes of Sca-1^+^ cells and lead to the discovery of novel molecular markers facilitating a better detection and functional evaluation. In this study, we isolated viable Sca-1^+^ and CD34^+^ cells on the basis of their protein expression. Following global amplification of single cell mRNA, gene expression profiling was performed to analyze the cell populations at the single cell level. The analysis included markers that have previously been ascribed to BASCs and intended to identify markers that thus far have not been described in Sca-1^+^ murine lung cells.

## Materials and Methods

### Animals and Tissue Preparation

No animal underwent animal experimentation as defined by the German law (§ 7 TierschG). All animals were euthanized and killed before organs were taken. According to German law (§ 6 Abs 1 TierschG) there is no requirement for an Ethics vote nor a notification of the local Government for dissection and organ use after the death of an animal. Mice were kept according to the guidelines of the Felasa (Federation for Laboratory Animal Science Associations) and the guideline 2010/63/EU and the ETS123 (APPENDIX A to the European Convention for the Protection of Vertebrate Animals used for Experimental and other Scientific Purposes).

Experiments were conducted on 26 Balb/c mice (10 female, 16 male) between 8 and 12 weeks of age. Mice were euthanized with 100% carbon dioxide, and subsequently the thoracic cavity was opened and the great venous vessels were clamped. The right ventricle was incised and a knop canula introduced to perfuse the lungs with 20–30 ml of body-warm saline. After exsanguination, lungs were dissected and collected in Hanks Balanced Salt Solution (HBSS, pH 7.4, Sigma Aldrich).

### Tissue Digestion and Cell Separation

The lungs were finely minced and the tissue was enzymatically digested according to an established protocol [13], with minor modifications. Briefly, each finely minced lung preparation was incubated with 5.4 U/ml collagenase (Roche), 0.03 U/ml dispase (GE Healthcare-Invitrogen) and 2.5 mM CaCl_2_ for 45 minutes at 37°C. The suspension was then filtered through nylon membranes (BD; 100 µm, 40 µm). Enzymes were inactivated in HBSS/0.2 M EDTA (pH 7.4), cells were centrifuged (1500 rpm, 4°C, 5 min) and resuspended in PBS (pH 7.4). A Percoll gradient (70%) density centrifugation was performed (2050 rpm, 4°C, 20 min) to separate the mononuclear cell fraction. The cell interphase was recovered and washed in PBS. Following a centrifugation step (1500 rpm, 4°C, 10 min) cells were resuspended in PBS (pH 7.4). Finally, viable cells were identified by Trypan blue assay and counted in a Neubauer chamber.

For FACS analysis, the lung preparations of 5 mice were finely minced. The tissue was enzymatically digested with collagenase (Sigma; 1.5 mg/ml) for 30 minutes at 37°C. The enzymes were inactivated with PBS/BSA (Sigma; 2.5 g/100 ml). The suspension was filtered through a cell strainer (Greiner Bio-One; 40 µm), centrifuged (300 g, 10 min, 4°C) and resuspended in 5 ml red blood cell lysis solution (1×) (Miltenyi; order no. 130-094-183) for 15 min at room temperature. Thereafter, cells were washed with PBS, centrifuged und resuspended in PBS to be counted in a Neubauer chamber.

### Immunofluorescence Staining and Single Cell Isolation

Immunofluorescence was performed on one million cells of each lung preparation with antibody concentrations of 10 µg/ml for all stainings. Cells were stained subsequently with either antibodies specific for CD31 (Biozol, clone 390) and Sca-1 (FITC-conjugated, Cedarlane, clone CT-6A/6E) or for CD34 (BD Pharmingen, clone RAM34) and CD45 (FITC-conjugated, Becton Dickinson GmbH, clone 30-F11). Cy3-conjugated secondary antibodies (Jackson ImmunoResearch Laboratories, code number: 115-166-071) were used to visualize CD31 or CD34 and Alexa 488-conjugated secondary antibodies (Molecular Probes, cat # A-11096) to visualize Sca-1 or CD45, respectively. Isotype controls were applied for all staining procedures as negative controls. Depending on the staining, Propidium iodide (PI, for Sca-1/CD31 staining) or GFP-Annexine (GFP-A, for CD34/CD45 staining) served as markers for apoptotic cells. Lung cells were screened on an 8-field glass slide for Sca-1^+^ and CD34^+^ cells (0.25×10^6^ cells/field) using an inverted fluorescence microscope (Zeiss, Germany) equipped with a micromanipulator (Eppendorf, Germany). Sca-1^+^/CD31^−/^PI^−^ and CD34^+^/CD45^−/^GFP-A^−^ cells were isolated and each single cell was separately transferred into a 0.2 ml reaction tube (1 cell/tube) containing 4 µl of lysis buffer and 0.4 µl of tRNA.

Since quantitative gene expression analysis of the isolated Sca-1^+^ and CD34^+^ cells was an important goal of our study, we had to acquire appropriate reference cells for comparison, i.e. pulmonary cells lacking Sca-1 and CD34 expression and not originating from hematopoietic or endothelial cell lines. Therefore, cells of 6 enzymatically digested lungs were double-stained with CD31 and CD45 antibodies in order to collect single cell samples. The same isolation procedure as described above was applied for CD31^−/^CD45^−^ reference cells. To exclude cells expressing Sca-1 and CD34, specific PCRs on the corresponding amplified transcripts of those genes were performed.

### Whole Transcriptome Amplification (WTA) of Single Cells

The transcriptome of each obtained cell was extracted and amplified according to an established protocol [12] with modifications [11]: One microlitre protease/lysis buffer mix (1∶20-dilution; Active Motif, Rixensart, Belgium) and 1 µl biotinylated peptide nucleic acids (PNAs; Midi-Kit, Active Motif, dissolved in 400 µl water) were added to each tube for proteolytic digestion. Digestion was performed at 45°C for 10 min, followed by inactivation for 1 min at 70°C and 15 min at 22°C for PNA annealing to the mRNA. mRNA was captured by streptavidine-coated magnetic particles (Active Motif) during 45 min rotation at room temperature. After addition of 10 µl wash buffer 1 (50 mM Tris-HCl (pH 8.3), 75 mM KCl, 3 mM MgCl_2_, 10 mM DTT and 0.25% Igepal), tubes were placed in a magnetic rack. The supernatants containing the genomic DNA were removed and the beads washed with 20 µl wash buffer 2 (50 mM Tris-HCl (pH 8.3), 75 mM KCl, 3 mM MgCl_2_, 10 mM DTT and 0.5% Tween). The supernatants were removed and the beads washed once again with 20 µl wash buffer 1. The mRNA was reverse transcribed using a mix comprising 0.5 mM dNTPs, 30 µM CFL15CN8 primer ([C]_n = 15_GTCTAGA[N_n = 8_]), 15 µM CFL15CT24 primer ([C]_n = 15_GTCTAGA[T]_n = 24_), 0.25% Igepal, 10 mM DTT (Invitrogen), the reaction buffer supplied by the manufacturer and 200 U of Superscript II reverse transcriptase (Invitrogen, Karlsruhe) in a final volume of 20 µl. Primers were allowed to anneal at room temperature for 10–15 min before the enzyme was added. Reverse transcription was conducted for 45 min at 44°C in an oven with constant steering. Following reverse transcription, the magnetic cDNA-mRNA-hybrids were washed in 20 µl tailing wash buffer (50 mM KH_2_PO_4_ (pH 7), 1 mM DTT, 0.25% Igepal), resuspended in 10 µl tailing buffer (10 mM KH_2_PO_4_ (pH 7), 4 mM MgCl_2_, 0.1 mM DTT, 200 µM dGTP) and then coated with 40 µl PCR oil. Hybrids underwent denaturation at 94°C for 4 min. The tailing reaction was performed at 37°C for 60 min after addition of 10 U terminal deoxynucleotide transferase (TdT; Amersham, Freiburg). TdT was inactivated at 70°C for 5 min. Next, a hot-start PCR was performed. Therefore, 35 µl of PCR mix 1 containing 4 µl buffer 1 (Expand long template, Roche) and 3% deionized formamide was added to each sample., Next, the samples were heat up to 78°C followed by addition of 5.5 µl of the PCR mix 2 (350 mM dNTPs, 1.2 µM CP2 primer (TCAGAATTCATG[C]_n = 15_) and 5 U Pol Mix (Expand long template)). Forty cycles were run in a MJ research PCR machine: 20 cycles of 15 sec at 94°C, 30 sec at 65°C, 2 min at 68°C and 20 cycles with an elongation of the extension time of 10 sec and a final elongation step of 7 min at 68°C.

### Analytical PCR on Specific Transcripts

For quality control of whole transcriptome amplification products, we tested for amplicons of ubiquitously expressed genes *Actb* (ß-actin) and *Gapdh* (Glyceraldehyde 3-phosphate dehydrogenase) by PCR. Only cells with at least one positive result were considered for further analysis. For initial molecular characterization of isolated cells, PCR on transcripts of *Sca-1*, *CD34*, *CD45* and *CD31* were performed.

In order to differentiate between a more epithelial or mesenchymal phenotype of isolated cells, we conducted further PCRs specific for epithelial markers *Epcam* (Epithelial cell adhesion molecule), *Itga* (Integrin alpha-6) and *Sftpc* (Surfactant protein C) and mesenchymal markers *CD90* (Thy-1) and *Pdgfrα* (platelet derived growth factor receptor alpha, CD140a), as suggested by McQualter et al. [9]. Specificity of all primers was confirmed by restriction digestion, sequences are depicted in Table S1.

### Array Hybridization and Data Analysis

Probes of the 29 selected cells were hybridized on Mouse Genome OpArrays (Eurofins MWG Operon; cat # OPMMV4-05). The arrays contain probes for 16,928 genes and have previously been used for hybridization of single cell WTA products [11]. The amplified single cell cDNA was labeled with 0.05 mM digoxygenin-dUTP (Roche) and 0.05 mM aminodigoxygenin-dCTP (PerkinElmer, Rodgau-Jügesheim) in the presence of 3% formamide, 2.4 µM CP2-BGL primer (TCAGAATTCATGCCGCCCCCCCGGCCC) and dNTPs (0.35 mM dATP and dGTP, 0.3 mM dTTP and dCTP). Reference cDNA was labeled with biotin-dUTP (Roche) and biotin-dCTP (Invitrogen). Primer sequences were then separated from the cDNA sequences in a subsequent digestion step with 30 U of BglI (Fermentas, St. Leon-Rot), and then purified (QIAquick PCR Purification Kit, QIAGEN, Hilden). Test and reference cDNA were co-precipitated with 0.8 µl polyacrylamide carrier, 0.1×3 M sodium acetate and 2.5× ethanol (100%). Arrays were pre-hybridized with 5× SSC +0.1% SDS +0.1% BSA at 42°C and hybridized in an Arraybooster hybstation (Implen, Munich) at 42°C overnight. The slides were washed twice at 42°C in 2× SSC+0.1% SDS for 5 min, twice at room temperature in 0.1× SSC +0.1% SDS for 10 min, and finally twice at room temperature in 0.1×SSC for 2 min 30 s. Unspecific binding of labeled proteins was blocked with 1% blocking reagent for nucleic acid hybridization (Roche). Slides were then stained with 16 µg/ml anti-Dig-Cy5 (Jackson Laboratories) and 18 µg/ml Streptavidin-Cy3 (Jackson Laboratories). In order to remove excess antibody/streptavidin, slides were washed with 4× SSC +0.2% Tween-20.

The microarray slides were scanned using the GenePix 4000A Arrayscanner (Molecular Device). The downstream data preprocessing and analysis was done in R using the limma package [14]. Raw probe intensities were background corrected by applying the ‘normexpr’ method. Analysis was restricted to Cy5 intensities. Loess normalization was used in M versus A plots of individual Cy5 intensities of a given transcript in a given array and the median Cy5 intensity across all arrays of the same transcript. Log2 ratios were calculated from the normalized intensities and quantile-normalization was subsequently applied across all arrays. All further analysis was based on normalized log ratios. Based on the hybridization date the data set is separated into two main batches of microarrays (early 2007/2008 and late 2009), which degrade further into small sub-batches. We used the ComBat algorithm [15] to adjust for the main batches. Transcripts differentially expressed between the groups were identified using regularized linear model as implemented in the limma package [16]. Transcripts were considered as significantly differentially expressed when their corresponding adjusted P-value was ≤0.05. Adjustment of P-values as correction for multiple testing was done as proposed by Benjamini et al. [17]. The differential gene expression analysis was used to generate candidates of regulated genes. The data have been deposited in NCBIs Gene Expression Omnibus (GEO, http://www.ncbi.nlm.nih.gov/geo/) and assigned series accession number GSE52215.

### Quantitative PCR

The differentially expressed genes for Decorin (*Dcn*), Gelsolin (*Gsn*) and Esterase D/formylglutathion hydrolase (*Esd*) were used to validate microarray results in an additional series of specific analytical and quantitative PCRs (qPCR). qPCR was performed using the LightCycler® 480 instrument (Roche, Mannheim, Germany). The real-time quantification was conducted using LightCycler® 480 SYBR Green I Master reagent (Roche, Mannheim, Germany). The cycling procedure consisted of an initial denaturation step (5 min at 95°C) followed by 38 cycles of 20 s at 95°C, 15 s at 58°C and 15 s at 72°C. All reactions were run in three replicas in a final volume of 19 µl containing 5 µl of cDNA template and 7 pmol of each forward and reverse primer. Positive and negative controls were included in all qPCR runs. Additionally, to confirm the specificity of reaction, all experiments included melt curve analysis. For each cell group three representative samples were generated by pooling equal amounts of single-cell cDNA samples assigned to a given group (10, 7 or 12 samples for *Sca-1^+^/CD34^+^*, *Sca-1^+^/CD34* ^−^ and reference cells, respectively). This was done to decrease the measurement noise originating from cell-to-cell variability of gene expression levels. 100× dilutions of the original cDNA sample representations were used as template for the qPCR. The relative gene expression levels were calculated using the efficiency corrected mathematical model described elsewhere [18]. Quantification was performed in parallel using three different reference genes *Actb*, *Gapdh* and *Hprt1* (Hypoxanthine phosphoribosyl transferase 1), in each case giving highly comparable results. Group-wise comparison of relative gene expression levels was performed using 2-tailed Student’s t-test. A value of p*<*0.05 was considered to indicate a statistically significant difference.

### FACS Staining

For FACS analysis a FACSCanto II (BD Biosciences) was used. Five million cells per animal were blocked with 1 µg Fc-block/tube and stained with anti-mouse CD31 antibody (PE-conjugated, clone 390; Biolegend, 1 µg/ml), anti-mouse CD34 antibody (FITC-conjugated, clone RAM34; eBioscience, 10 µg/ml), anti-mouse CD45 antibody (PerCP/Cy5.5-conjugated, clone 30-F11; Biolegend, 2.5 µg/ml), anti-mouse Sca-1 antibody (APC-Cy7-conjugated, clone D7; Biolegend, 2.5 µg/ml), anti-mouse EpCAM antibody (eF450-conjugated, clone G8.8; eBioscience, 10 µg/ml) and anti-mouse PDGFRα antibody (APC-conjugated, clone APA5; Biolegend, 10 µg/ml) or corresponding isotype antibodies for 20 min at 4°C. Results were visualized by the FlowJo package (Tree Star Inc., Ashland, OR, USA).

## Results

### Identification of Sca-1^+^ and CD34^+^ Cells and Selection for Molecular Analysis

**Figure 1:**
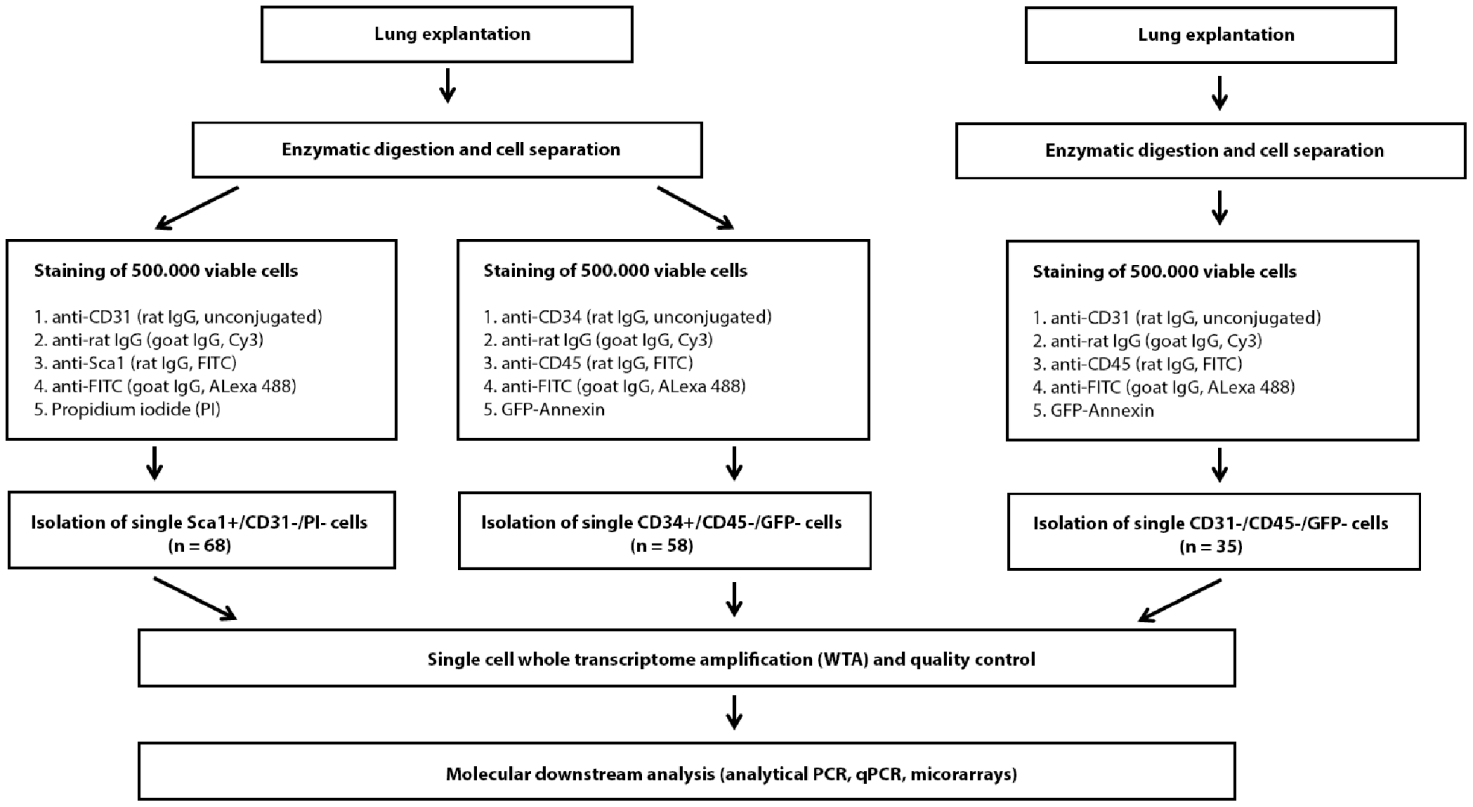
Flowchart of the experimental setup. Simplified schematic overview on the workflow of single cell isolation and immunofluorescent staining strategy: Cells are divided into two groups after separation and stained with antibodies directed against CD31 and Sca-1 or CD34 and CD45, respectively. In order to gain appropriate reference cells for comparative gene expression analysis, additional lung cells are stained with antibodies directed against CD31 and CD45, and only CD31^−/^CD45^−^ cells are isolated. Propidium iodide (PI) and GFP-Annexine (GFP-A) are applied to exclude apoptotic cells. Genes recently introduced in literature in order to differentiate between epithelial and mesenchymal cells are tested by specific PCR. PCR results enable further subdivision of analyzed cells (Sca-1^+^/CD34^+^ cells, Sca-1^+^/CD34^−^ cells, Sca-1^−/^CD34^+^ cells). In a final step, selected Sca-1^+^/CD34^+^ cells, Sca-1^+^/CD34^−^ cells and Sca-1^−/^CD34^−^ reference cells are subjected to comparative gene expression analysis. Results are validated by qPCR of pooled samples.

In order to isolate Sca-1^+^ and CD34^+^ cells 15 Balb/c mice between 8 and 12 weeks of age were sacrificed. Tissue digestion and cell separation started immediately after lung explantation. The number of obtained non-erythrocytic, non-apoptotic cells per experiment ranged from 0.40×10^6^ to 5.0×10^6^ (mean 1.6×10^6^; standard error of the mean (SEM) 1.16×10^6^). The number of erythrocytes ranged from 0.20×10^6^ to 2.6×10^6^ (mean 0.87×10^6^; SEM 0.73×10^6^), the number of apoptotic cells (i.e. PI^+^ or GFP-A^+^ cells) ranged from 0.92×10^6^ to 4.0×10^6^ (mean 1.9×10^6^; SEM 1.3×10^6^). The experimental setup is outlined in Figure 1.

Up to one million cells per animal were screened, equally divided for Sca-1/CD31 and CD34/CD45 immunofluorescence (i.e. up to 0.50×10^6^ cells for each staining and animal). We identified and isolated 68 Sca-1^+^/CD31^−/^PI^−^ cells, the number of obtained cells per mouse ranging from 0 to 10 (mean 4.5, SEM 3.0, Table 1 and Figure S1). Additionally, we detected 58 CD34^+^/CD45^−/^GFP-A^−^ cells, ranging from 0 to 13 in individual mice (mean 3.9; SEM 4.6, Table 1 and Figure S1). All detected cells were isolated and subjected to single cell gene expression analysis following whole transcriptome amplification (WTA). The enzymatically digested lungs of 6 additional mice yielding up to 1.7×10^6^ non-erythrocytic cells (mean 1.3×10^6^ cells; SEM 0.32×10^6^ cells) were stained with antibodies against CD31 and CD45 resulting in the isolation of 35 control cells (CD31^−/^CD45^−^). All isolated cells were subjected to whole transcriptome amplification (WTA) and WTA quality controlled by expression of *Actb* and *Gapdh*. In total, 115/161 (71.4%) samples passed our quality assay and were subjected to further molecular analysis (no difference between cell types, data not shown).

**Table 1:** Non-erythrocytic cells, Sca-1^+^ and CD34^+^ cells per lung preparation.

Mice	non-erythrocyticcells	Sca-1^+^/CD31^−^cells	CD34^+^/CD45^−^cells
1	680000	3	0
2	1.52×10^6^	4	3
3	920000	4	2
4	1.2×10^6^	4	5
5	1.2×10^6^	0	0
6	2.4×10^6^	5	6
7	2.0×10^6^	5	11
8	1.2×10^6^	6	12
9	2.4×10^6^	3	3
10	400000	3	1
11	520000	3	0
12	1.2×10^6^	8	2
13	1.0×10^6^	0	13
14	5.0×10^6^	10	0
15	2.5×10^6^	10	0

We continued by testing for the presence of transcripts of the proteins targeted by antibodies in immunofluorescence: *Sca-1, CD34, CD45 and CD31*. Within the two populations of putative BASC, 35 cells completely lacked transcript expression of *Sca-1*/*CD34*, while another 10 cells co-expressed *CD31* and/or *CD45* (Table 2). We decided to exclude those cells from further analyses which resulted in a cohort of 46 single putative BASCs remaining for downstream analyses. Likewise, among the tested pulmonary reference cells we excluded one sample expressing *Sca-1* and two samples positively tested for the presence of *CD45* transcripts resulting in a cohort of 21 cDNA libraries of *Sca-1^−/^CD34* ^−^ cells.

**Table 2:** PCR results of corresponding transcripts in Sca-1+/CD31- and cells CD34+/CD45- cells.

PCR result				Immunofluorescence	
*Sca-1*	*CD31*	*CD34*	*CD45*	Sca-1^+^/CD31^−/^PI^−^	CD34^+^/CD45^−/^GFP-A^−^
				N	N
**+**	**–**	**–**	**–**	15	7
**+**	**-**	**+**	**–**	4	13
**-**	**-**	**+**	**–**	5	2
**+**	**-**	**+**	**+**	2	1
**+**	**–**	**–**	**+**	3	0
**–**	**–**	**+**	**+**	2	1
**–**	**–**	**–**	**+**	12	10
**–**	**+**	**–**	**–**	1	0
**–**	**–**	**–**	**–**	7	3
**+**	**+**	**–**	**+**	0	1
**–**	**+**	**–**	**+**	0	2

### Correlation between Protein and mRNA Expression in Single Cells

Next, we assessed the correlation between protein staining and PCR results for *Sca-1* and *CD34* in the group of putative BASCs (Table 3). In total, 24/46 cells were isolated as Sca-1^+^/CD31^−/^PI^−^ and 22/46 cells as CD34^+^/CD45^−/^GFP-A^−^ using immunofluorescent staining (Figure 1). Direct comparison revealed that Sca-1 expression could be detected simultaneously at both protein and mRNA level in 19 of 24 Sca-1^+^/CD31^−/^PI^−^ cells (79.2%) and *CD34* expression could be detected on protein and mRNA level in 15 of 22 CD34^+^/CD45^−/^GFP-A^−^ cells (68.2%), thus showing a positive correlation between protein and transcript level in the majority of putative BASCs. According to the detected mRNA transcripts after single cell WTA, cells could be grouped either as *Sca-1^+^/CD34^+^* (n = 17), *Sca-1^+^/CD34* ^−^ (n = 22) or *Sca-1^−/^CD34^+^* (n = 7).

**Table 3:** Distribution of PCR-based *Sca-1/CD34* expression in isolated putative BASCs.

		Immunofluorescence		
		Sca-1^+^	CD34^+^	
**mRNA transcripts**	*Sca-1^+^/CD34^+^*	4	13	17
	*Sca-1^+^/CD34* ^−^	15	7	22
	*Sca-1^−/^CD34^+^*	5	2	7
		24	22	46

Interestingly, simultaneous expression of Sca-1 and CD34 could be detected in 13/22 single cells isolated after CD34-staining (59.1%) and only in 4/24 (16.7%) single cells isolated after Sca-1-staining, resulting in a significantly higher prevalence of cells showing mRNA transcripts of both markers Sca-1 and CD34 in the group of CD34^+^/CD45^−/^GFP-A^−^ cells (Fisher’s exact test, p = 0.005, Table 3). On the other hand, the group of Sca-1^+^/CD31^−/^PI^−^ cells showed a higher prevalence for cells positive for *Sca-1* transcripts only, an expression pattern that matched 15/24 Sca-1^+^/CD31^−/^PI^−^ cells and 7/22 and CD34^+^/CD45^−/^GFP-A^−^ cells, respectively (Chi Square test, p = 0.04, Table 3). These results indicate the existence of different subpopulations within the isolated fractions of cells.

### Identification of Novel Molecular Markers in Putative BASCs

To further analyze the isolated cells, we selected 17 putative BASCs (10 *Sca-1^+^/CD34^+^* cells and 7 *Sca-1^+^/CD34* ^−^ cells) and 12 reference cells (*Sca-1^−/^CD34^−/^CD31^−/^CD45* ^−^ *)* for hybridization on Mouse Genome OpArrays (Eurofins MWG Operon). Here, we decided to compare microarray data of two of the three cell groups independently with each other.

**Figure 2:**
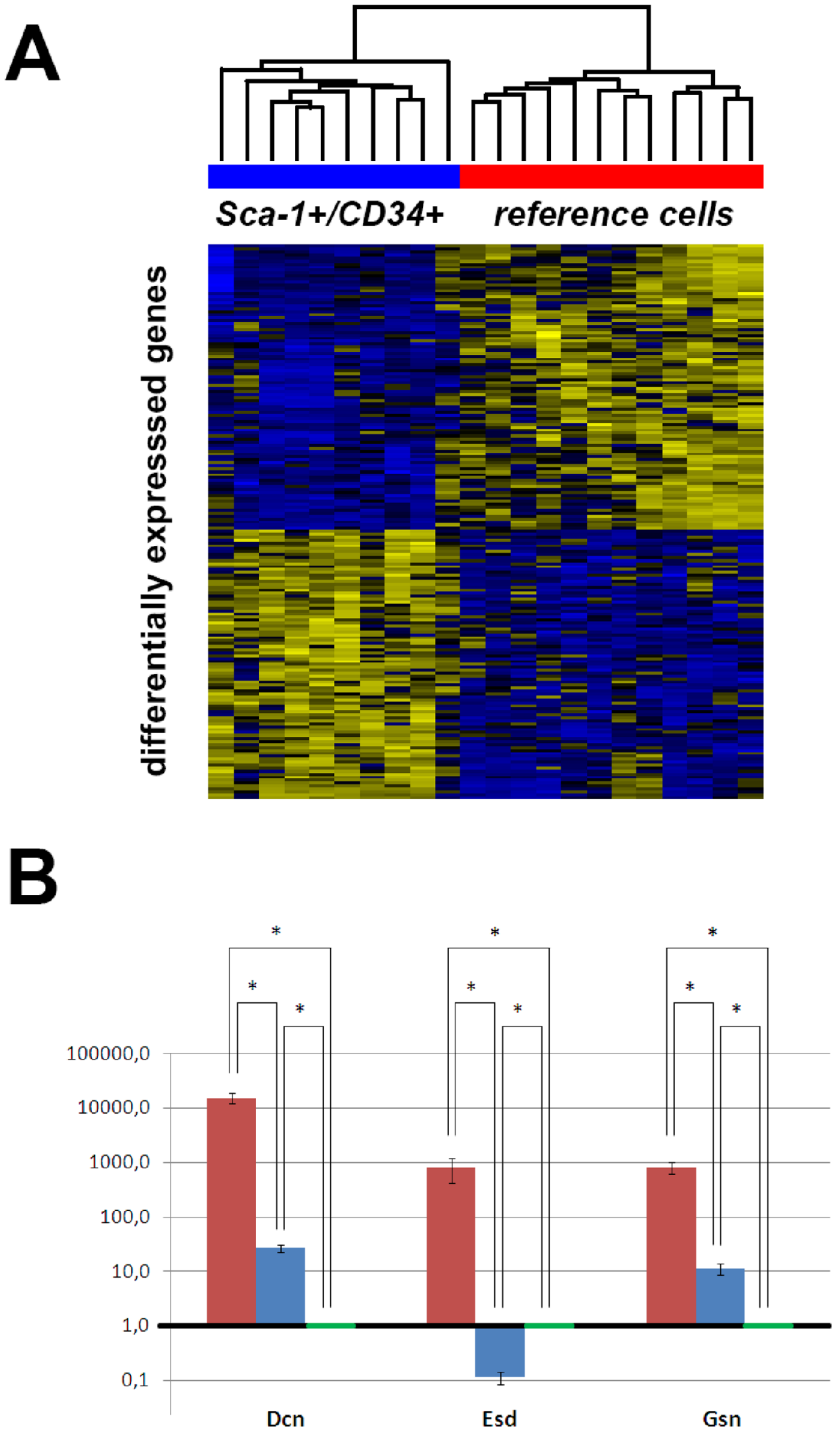
Gene expression microarray analysis of isolated single cells. **Panel A:** The comparison of microarray expression profiles of *Sca-1^+^/CD34^+^* cells and pulmonary reference (Sca-1^−/^CD34^−/^CD31^−/^CD45^−^) cells showed 107 differentially expressed genes. **Panel B:** Pools of analyzed cells from *Sca-1^+^/CD34^+^* subpopulation (red), *Sca-1^+^/CD34* ^−^ subpopulation (blue) and pulmonary reference cells (green) were analyzed by quantitative PCR against differentially expressed genes *Dcn*, *Esd* and *Gsn*. The selected genes not only show significant differences regarding to their expression, but also represent different subpopulations of proteins. Error bars indicate standard deviation of the mean calculated for analyzed triplicates. Expression values are calculated by relative quantification against housekeeping gene *Actb* and illustrated in comparison to pulmonary reference cells (expression value = 1.0) on a logarithmic scale. All comparisons between different groups, as determined by quantitative PCR, showed significantly different expression levels (student’s t-test, * indicating p<0.05).

First, we analysed data of *Sca-1^+^/CD34^+^* cells and the selected pulmonary reference cells only, which resulted in detection of significant changes in expression levels of 107 genes (adjusted p-value <0.05 each, Figure 2A, Table S2). Interestingly, we could not find any differentially expressed genes between the groups of *Sca-1^+^/CD34* ^−^ cells and the reference cells (*Sca-1^−/^CD34* ^−^). In turn, comparative gene expression analysis of *Sca-1^+^/CD34^+^* and *Sca-1^+^/CD34* ^−^ cells identified 8 differentially expressed genes (adjusted p-value <0.05 each, Table S3).

In general, gene expression in single cells is subjected to stochastic fluctuations [19]. While in high-dimensional data (as microarrays) normalization generally uses many if not all probes, relative quantification in quantitative PCR (qPCR) experiments relies on stable expression levels of individual genes. This especially holds true for some house-keeping genes, as e.g. *Actb* or *Gapdh*, which renders reliable relative quantification using such an approach at the single cell level questionable [20], [21]. Although specific genes can be expressed at a stable level between individual single cells of a certain cell type, this approach is not feasible for poorly described cell populations. Therefore, we decided to pool the single cell samples of a pre-defined subgroup for qPCR analyses to validate the results of the microarray analyses.

For validation, we chose transcripts for Decorin (*Dcn*), and Gelsolin (*Gsn*), which were differentially expressed between *Sca-1^+^/CD34^+^* and reference cells, as well as Esterase D/formylglutathion hydrolase (*Esd*), which was in addition differentially expressed between *Sca-1^+^/CD34^+^* and *Sca-1^+^/CD34* ^−^ cells. Besides the differential expression, the rationale for the selection of the three chosen transcripts was that they represent different functional groups of genes. As expected, cells from the *Sca-1^+^/CD34^+^* subpopulation expressed very high levels of all three transcripts in comparison to cells of the other two groups (student’s t-test, p<0.05). If comparing *Sca-1^+^/CD34* ^−^ cells with pulmonary reference samples, Dcn and Gsn were expressed in significantly higher levels in *Sca-1^+^/CD34* ^−^ cells than *Sca-1^−/^CD34* ^−^ cells, while Esd was expressed at significantly lower level in *Sca-1^+^/CD34* ^−^ cells than in pulmonary reference cells as determined by quantitative PCR (student’s t-test, p<0.05, Figure 2B).

### Single Cell Analysis of Epithelial and Mesenchymal Transcripts Allows Further Delineation of Sca-1^+^/CD34^+,−^ Subpopulations

To further clarify the epithelial or mesenchymal commitment of isolated cells, we checked expression of mesenchymal (*CD90*, *Pdgfrα*) and epithelial transcripts (*Epcam*, *Itga* and *Sftpc*) as recently described [8]. For this purpose, we analyzed all 46 putative BASCs and 21 pulmonary reference cells by analytical PCR (Table S4).

**Figure 3:**
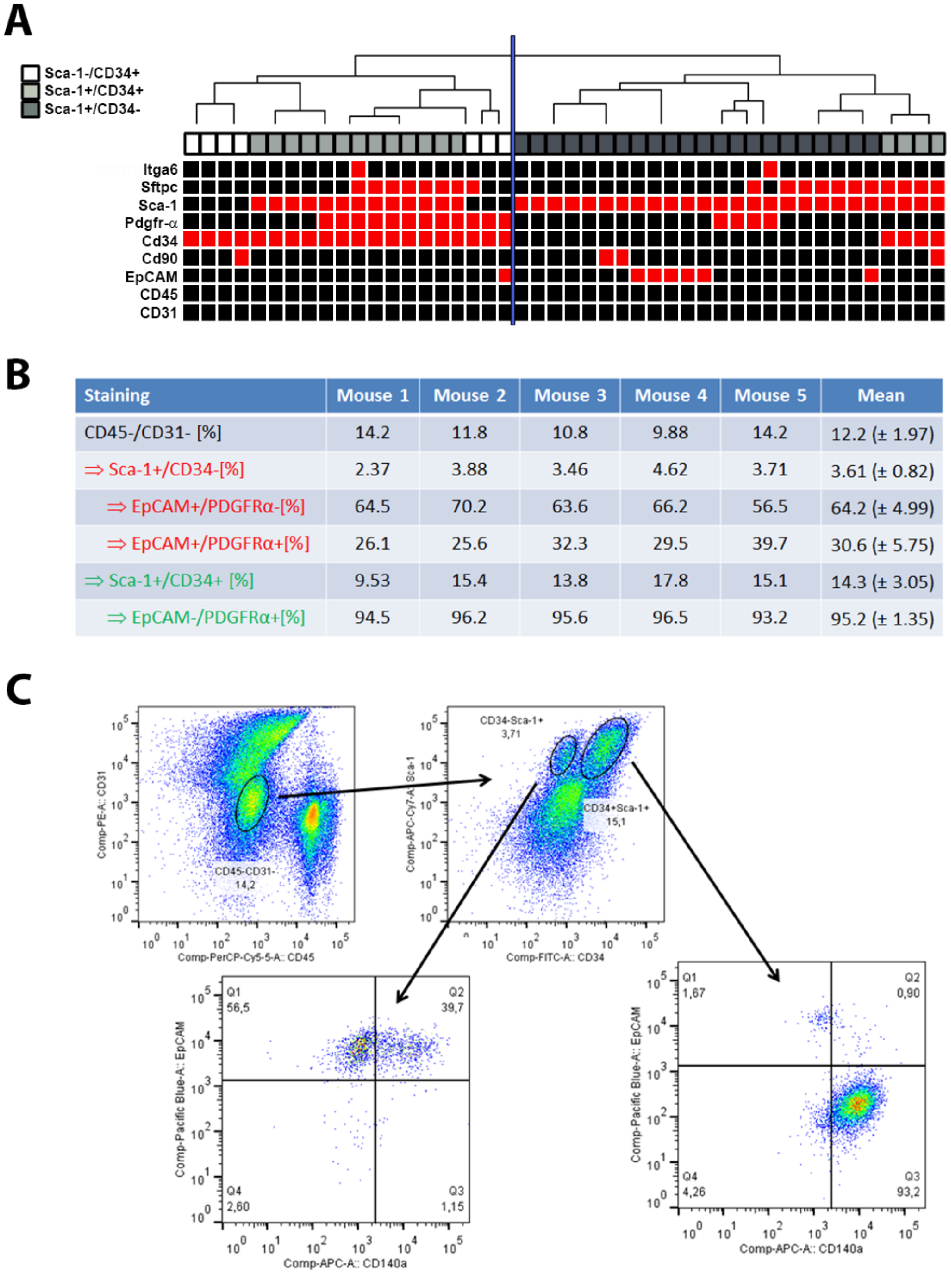
Expression of epithelial and mesenchymal markers in *Sca-1^+^/CD34^+,^^−^* cells. **Panel A:** The expression of epithelial and mesenchymal transcripts was tested by analytical PCR and is illustrated in a hierarchical cluster heatmap. The analysis shows that the majority of *Sca-1^+^/CD34^+^* cells (light grey) show similar marker expression as *Sca-1^−/^CD34^+^* cells (white), while all *Sca-1^+^/CD34* ^−^ cells (dark grey) are located in the second branch. Red squares indicate specific bands in analytical PCR, black squares indicate negative PCR results. **Panel B:** FACS analysis reveals EpCAM^+^/Pdgfrα^+^ subpopulation within Sca-1^+^/CD34^−/^CD31^−/^CD45^−^ cells. For each of 5 mice, 5×10^6^ murine lung cells were isolated from lung explants and stained with antibodies directed against Sca-1, CD34, CD31, CD45, Pdgfrα and Epcam. While Sca-1^+^/CD34^−^ cells consistently showed Epcam expression, Pdgfrα expression was predominantly found in Sca-1^+^/CD34^+^ cells. However, Sca-1^+^/CD34^−/^Epcam^+^ cells could be divided in two major subpopulations defined by Pdgfrα expression. Relative quantification is given for corresponding selected subpopulation as indicated by arrows. **Panel C:** Scatter plots of the detected cell populations for mouse 5, only.

While statistical analysis revealed no differences in the expression frequency of mesenchymal markers in the group of putative BASCs compared to reference cells, significant differences were detected between Sca-1^+^/CD34^−^ cells (n = 22) and Sca-1^+^/CD34^+^ cells (n = 17, Figure 3A). Here, we noted that the epithelial transcript *Epcam* was exclusively expressed in some Sca-1^+^/CD34^−^ cells (6/22 cells; p = 0.03, Fisher’s exact test). In comparison, the mesenchymal transcript *Pdgfrα* was more frequently expressed by *Sca-1^+^/CD34^+^* cells (9/17 cells), although 4/22 *Sca-1^+^/CD34* ^−^ cells also expressed transcripts of the gene (p = 0.04, Fisher’s exact test). Interestingly, while the putative epithelial marker *Sftpc* was frequently expressed in both major cell types, it could be detected more frequently in *Sca-1^+^/CD34^+^* than *Sca-1^+^/CD34* ^−^ cells (11/17 vs. 7/22 single cells; p = 0.04, Chi-square test). *Itga* and *CD90* were rarely expressed in single cells of either cell type. Within the small group of *Sca-1^−/^CD34^+^* cells, 2/7 cells co-expressed epithelial and mesenchymal markers, whereas another 2/7 cells expressed mesenchymal markers only (Table S4). The remaining three cells were negative for both mesenchymal and epithelial markers.

In order to confirm the PCR results we performed FACS analysis of 5 additional digested lung explants using antibodies directed against Sca-1, CD34, CD45, CD31, Epcam and Pdgfrα. Within the population of CD45^−/^CD31^−^ cells, we could detect 3.61% of Sca-1^+^/CD34^−^ cells and 14.3% of Sca-1^+^/CD34^+^ cells in mean (Figure 3B). We proceeded to look at Epcam and Pdgfrα expression in these two subpopulations of putative BASCs. As previously described, the majority of Sca-1^+^/CD34^−^ cells expressed Epcam, while Pdgfrα was expressed in the Sca-1^+^/CD34^+^ subpopulation. However, in good correlation to our PCR results, we found a significant subpopulation of Epcam^+^/Pdgfrα^+^ cells within the subpopulation of Sca-1^+^/CD34^−^ cells (Figure 3B and C).

## Discussion

In this study we investigated the gene expression profile of single Sca-1^+^/CD31^−/^PI^−^ and CD34^+^/CD45^−/^GFP-A^−^ cells that were detected by immunofluorescence and subsequently sub-divided into groups based on the protein expression of Sca-1 and CD34, as well as mRNA expression of subgroup-specific markers.

Sca-1 and CD34 have both been ascribed to bronchioalveolar stem cells (BASCs), a rare population of long-living, regional fixed, robust cells residing at the bronchioalveolar duct junction that have been sparsely investigated so far. Only few markers were established in previous studies mainly applying FACS analysis, which enables a rapid screening of very large cell numbers and subsequent comparison between cell populations as defined by applied protein markers. This approach shows high sensitivity and at the same time an elevated risk to isolate false-positive cells, which may impair the detection of extremely rare cells [22]. In this study, we intentionally utilized a microscope-based approach to isolate single cells to analyze cell-to-cell heterogeneity in poorly characterized cells. Considering the low incidence of putative BASCs in lung tissue, we believe that this approach is the method of choice for the molecular analysis of such extremely rare and poorly characterized cells. Beyond that, our workflow additionally allows direct assessment of morphology and viability of the target cells, thereby reducing the risk of contamination with false-positive cells or cell debris to an absolute minimum.

Combined protein and cDNA analysis of Sca-1 and CD34 in our study showed a highly positive correlation between staining and transcript detection (79.2% for Sca-1 and 68.2% for CD34, respectively). In addition, our approach enabled further classification in different subpopulations characterized by *Sca-1* and *CD34* transcript expression. Analyzed cells consisted of two larger groups of *Sca-1^+^/CD34* ^−^ (n = 22) and *Sca-1^+^/CD34^+^* (n = 17) cells and a smaller group of *Sca-1^−/^CD34^+^* (n = 7) cells.

To further investigate the expression profiles of the different cell types, we performed microarray-based gene expression analysis of single cell WTA products, including 10 *Sca-1^+^/CD34^+^* cells, 7 *Sca-1^+^/CD34* ^−^ cells, and 12 *Sca-1^−/^CD34* ^−^ pulmonary reference cells. Comparisons between the different subgroups revealed 107 genes differentially expressed in *Sca-1^+^/CD34^+^* cells when compared to the reference cells, whereas no differentially expressed genes could be detected by comparing *Sca-1^+^/CD34* ^−^ cells with reference cells. The comparison between the two *Sca-1^+^* cell subgroups (*Sca-1^+^/CD34^+^* vs. *Sca-1^+^/CD34* ^−^) yielded 8 differentially expressed genes. Validation by quantitative PCRs for the genes *Dcn*, *Gsn* and *Esd* confirmed the microarray results.

During the validation of the microarray results we intentionally abstained from performing qPCR analysis directly on single cell WTA products due to stochastic variation in expression level, a phenomenon that has been repeatedly reported before [19], [23]–[25]. Cell-to-cell heterogeneity in gene expression, which is also detectable in our data sets, may represent stochastic transcriptional bursts that are generated by intrinsic on-off transitions of the corresponding genes occurring at irregular intervals [23], [25]. Fluctuations in levels of individual transcripts also affect the housekeeping genes, whose expression serves as reference in qPCR analyses [20], [21]. Stable reference is indispensable to reliably quantify relative expression levels of genes of interest. Higher stability in gene expression levels facilitating more reliable downstream analysis can be achieved only by pooling single cell samples representing the same cell population, thereby averaging the stochastic variability of transcript levels in individual cells especially for reference genes.

We selected three genes from the list of differentially expressed genes for qPCR validation coding for proteins of different functional groups (*Dcn*, *Gsn*, and *Esd*), representing extracellular matrix components, intracellular/membrane-bound proteins and cytoplasmic metabolic proteins, respectively. Antiproliferative or tissue stabilizing effects as well as the promotion of cellular motility and invasion have been demonstrated for Gelsolin and Decorin [26]–[31]. Both markers have been previously assigned to pulmonary fibroblasts. Consequently, their enhanced expression in Sca-1^+^/CD34^+^ cells would be in line with mesenchymal commitment. On the other hand, an exclusive expression of the novel markers in mesenchymal cells has not been proven so far. *Esd* has been ascribed an important role in intracellular detoxification [32] making tumor cells more resistant against harmful substances [33].

We then searched for transcripts of recently described epithelial (*Epcam*, *Itga*, *Sftpc*) and mesenchymal (*CD90*, *Pdgfrα*) markers [8] by analytical PCRs and found a frequent expression of *Pdgfrα* in *Sca-1^+^/CD34^+^* single cells. Interestingly, almost 80% of these cells additionally showed expression of *Sftpc*, described as an epithelial marker, while only 14% of *Sftpc*-expressing *Sca-1^+^/CD34* ^−^ cells showed coexpression of *Pdgfrα*. Moreover, expression of the epithelial transcript *Epcam* was restricted to the group of *Sca-1^+^/CD34* ^−^ cells (and one *Sca-1^−/^CD34^+^* cells), while expression of the mesenchymal marker *Pdgfrα* could be detected in only four of the analyzed *Sca-1^+^/CD34* ^−^ cells. In FACS analysis, we could confirm a significant subpopulation of Epcam+/Pdgfrα+ cells within the Sca-1+/CD34−/CD45−/CD31- cells (Figure 3).

Our results support an epithelial commitment of *Sca-1^+^/CD34* ^−^ cells – in contrast to the *Sca-1^+^/CD34^+^* cells that displayed an increased incidence of mesenchymal marker expression. However, taking into account the novel subpopulation of Sca-1^+^/CD34^−/^EpCAM^+^/Pdgfra^+^ cells we postulate that single cell isolation and transcription profiling on rare cells represents a sensitive approach to obtain molecular data of single cells of interest. In this study, we showed on single murine lung cells that this approach has the power to deliver novel molecular markers that could contribute to a better understanding of the cellular heterogeneity and hierarchy in the murine lung. The presented approach may, therefore, help to analyze extremely rare and yet poorly characterized cells (e.g. stem cells) in other fields of biology as well.

## Supporting Information

Figure S1 **Immunofluorescence stained single cell suspensions of explanted lungs. Panels A–D:** The arrow points to a single CD34^+^/CD45^−/^GFP-Annexin^−^ cell (Cy3-signal) surrounded by several CD34-negative cells showing fluorescence in FITC channel. **Panels E–F:** Single Sca-1^+^/CD31^−/^PI^−^ cell.
(TIF)

Table S1 **Primer sequences.**
(DOC)

Table S2 **Differentially expressed genes (Sca1+/CD34+ vs Reference cells).**
(DOC)

Table S3 **Differentially expressed genes (Sca1+/CD34+ vs Sca1+/CD34–).**
(DOC)

Table S4 **Detected mRNA transcripts in isolated single cells.**
(DOC)
