# Supplementary figures and images for: Molecular Profiling of Single Sca-1+/CD34+,− Cells—The Putative Murine Lung Stem Cells
Source: PLoS One. 2013 Dec 31;8(12):e83917. doi: 10.1371/journal.pone.0083917 (PMC3877111; doi:10.1371/journal.pone.0083917)

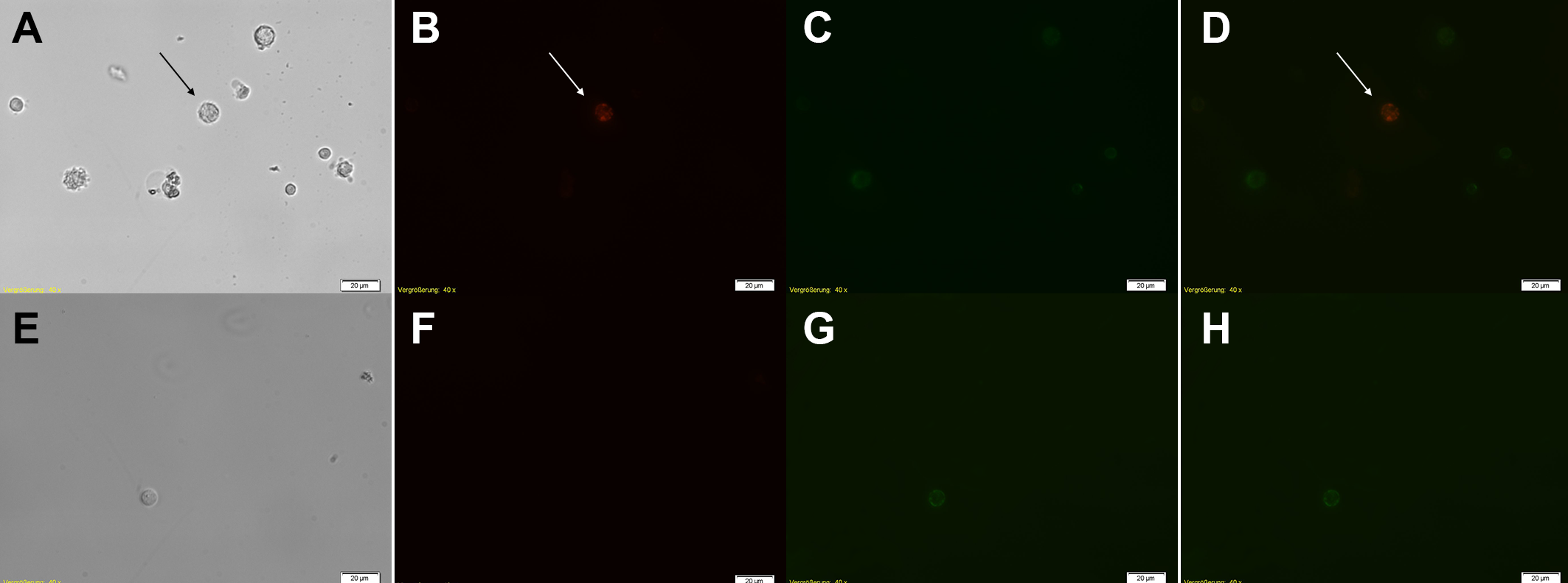

Supplement: Figure S1 — Immunofluorescence stained single cell suspensions of explanted lungs. Panels A–D: The arrow points to a single CD34+/CD45−/GFP-Annexin− cell (Cy3-signal) surrounded by several CD34-negative cells showing fluorescence in FITC channel. Panels E–F: Single Sca-1+/CD31−/PI− cell. (TIF) [file pone.0083917.s001.tif]
