# Supplementary material for: Molecular Profiling of Single Sca-1+/CD34+,− Cells—The Putative Murine Lung Stem Cells
Source: PLoS One. 2013 Dec 31;8(12):e83917. doi: 10.1371/journal.pone.0083917 (PMC3877111; doi:10.1371/journal.pone.0083917)
Supplement: Table S1 — Primer sequences. (DOC) [file pone.0083917.s002.doc]

**Table S**1: Primer sequences

| **Gene** | **Orientation** | **Sequence** | **Annealing Temperature** | **Fragment Size** |
| --- | --- | --- | --- | --- |
| *Actb* | forward | GTGACAGCATTGCTTCTGTG | 58°C | 214 bp |
|  | reverse | TCTCAAGTCAGTGTACAGGC |  |  |
| *Gapdh* | forward | GCTGAGTATGTCGTGGAGTC | 58°C | 193 bp |
|  | reverse | AGTTGGTGGTGCAGGATGC |  |  |
| *Sca-1* | forward | AGCTCAGTCCTGCAGA | 58°C | 150 bp |
|  | reverse | TTTCACACACTACTCCCACC |  |  |
| *CD34* | forward | AGGCTGATGCTGGTGCTAG | 58°C | 209 bp |
|  | reverse | AGTCTTTCGGGAATAGCTCTG |  |  |
| *CD45* | forward | TTGTCTGAGTTACATTCATGCCTAC | 52°C | 219 bp |
|  | reverse | GCTCATCTCCAGTTCATGCT |  |  |
| *CD31* | forward | GGTGGTTGTCATTGGAGTGG | 59°C | 182 bp |
|  | reverse | GAAGCAGCACTCTTGCAGTC |  |  |
| *Epcam* | forward | GAGTCCGAAGAACCGACAAG | 60°C | 127 bp |
|  | reverse | GCAGTCTGCAAGCTCTGATG |  |  |
| *Itga6* | forward | agccccagggacttacaact | 60°C | 230 bp |
|  | reverse | ctcttggagcaccagacaca |  |  |
| *Sftpc* | forward | cagctccaggaacctactgc | 60°C | 200 bp |
|  | reverse | cacagcaaggcctaggaaag |  |  |
| *CD90* | forward | TCCAGAATCCAAGTCGGAAC | 58°C | 159 bp |
|  | reverse | GTTATTCTCATGGCGGCAGT |  |  |
| *Pdgfrα* | forward | tggcatgatggtcgattcta | 60°C | 152 bp |
|  | reverse | cgctgaggtggtagaaggag |  |  |
| *Dcn* | forward | TGAGCTTCAACAGCATCACC | 59°C | 181 bp |
|  | reverse | AAGTCATTTTGCCCAACTGC |  |  |
| *Gsn* | forward | CTGGGCGGGAAGACTGCCTA | 59°C | 294 bp |
|  | reverse | GGTCCGCCTGTCCCGATTTG |  |  |
| *Esd* | forward | TCTTCCCTGGCGAGTTAGAA | 58°C | 158 bp |
|  | reverse | GGGGTGGTAGGTAGACAGCA |  |  |
