# Supplementary material for: Molecular Profiling of Single Sca-1+/CD34+,− Cells—The Putative Murine Lung Stem Cells
Source: PLoS One. 2013 Dec 31;8(12):e83917. doi: 10.1371/journal.pone.0083917 (PMC3877111; doi:10.1371/journal.pone.0083917)
Supplement: Table S2 — Differentially expressed genes (Sca1+/CD34+ vs Reference cells). (DOC) [file pone.0083917.s003.doc]

**Table S2: Differentially expressed genes (Sca1+/CD34+ vs Reference cells)**

|  | **Gene symbol** | **Fold change** | **Adj. P-value** | **Gene description** |
| --- | --- | --- | --- | --- |
| 1 | Dcn | 4.6786 | 0.00047633 | decorin [Source:MarkerSymbol;Acc:MGI:94872] |
| 2 | Esd | 2.6990 | 0.00100194 | esterase D/formylglutathione hydrolase [Source:MarkerSymbol;Acc:MGI:95421] |
| 3 | Ercc2 | -2.0080 | 0.00667724 | excision repair cross-complementing rodent repair deficiency, complementation group 2 [Source:MarkerSymbol;Acc:MGI:95413] |
| 4 | Egr1 | 3.4020 | 0.00735446 | early growth response 1 [Source:MarkerSymbol;Acc:MGI:95295] |
| 5 | Tgs1 | -1.6841 | 0.00878276 | trimethylguanosine synthase homolog (S. cerevisiae) [Source:MarkerSymbol;Acc:MGI:2151797] |
| 6 | Kbtbd10 | 3.3601 | 0.01044674 | kelch repeat and BTB (POZ) domain containing 10 [Source:MarkerSymbol;Acc:MGI:2683854] |
| 7 | Gsn | 4.0369 | 0.01045801 | gelsolin [Source:MarkerSymbol;Acc:MGI:95851] |
| 8 | Exoc6b | 1.5465 | 0.0117516 | exocyst complex component 6B [Source:MarkerSymbol;Acc:MGI:1923164] |
| 9 | B930008K04Rik | -1.5418 | 0.01525561 | RIKEN cDNA B930008K04 gene [Source:MarkerSymbol;Acc:MGI:2442284] |
| 10 | 2310020F24Rik | 1.4728 | 0.01580445 | RIKEN cDNA 2310020F24 gene [Source:MarkerSymbol;Acc:MGI:1921503] |
| 11 | Slc9a2 | 1.5354 | 0.01944117 | solute carrier family 9 (sodium/hydrogen exchanger), member 2 [Source:MarkerSymbol;Acc:MGI:105075] |
| 12 | 9230110F15Rik | 1.7804 | 0.01944117 | RIKEN cDNA 9230110F15 gene [Source:MarkerSymbol;Acc:MGI:1924330] |
| 13 | Ccdc66 | 1.6679 | 0.01944117 | coiled-coil domain containing 66 [Source:MarkerSymbol;Acc:MGI:2443639] |
| 14 | Mmp2 | 2.8586 | 0.01944117 | matrix metallopeptidase 2 [Source:MarkerSymbol;Acc:MGI:97009] |
| 15 | Cyr61 | 2.7561 | 0.01944117 | cysteine rich protein 61 [Source:MarkerSymbol;Acc:MGI:88613] |
| 16 | Fsip1 | -1.9593 | 0.01944117 | fibrous sheath-interacting protein 1 [Source:MarkerSymbol;Acc:MGI:1918563] |
| 17 | NP_001032841.1 | 2.0553 | 0.01944117 | beta-defensin 27 [Source:RefSeq_peptide;Acc:NP_001032841] |
| 18 | Igsf4d | 1.6527 | 0.01944117 | immunoglobulin superfamily, member 4 [Source:MarkerSymbol;Acc:MGI:2442722] |
| 19 | Best3 | -1.5317 | 0.01944117 | bestrophin 3 [Source:MarkerSymbol;Acc:MGI:3580298] |
| 20 | Chka | -1.3477 | 0.01944117 | choline kinase alpha [Source:MarkerSymbol;Acc:MGI:107760] |
| 21 | OTTMUSG00000005421 | -1.4793 | 0.02040692 | predicted gene, OTTMUSG00000005421 [Source:MarkerSymbol;Acc:MGI:3651509] |
| 22 | F730031O20Rik | -1.7183 | 0.02072896 | RIKEN cDNA F730031O20 gene [Source:MarkerSymbol;Acc:MGI:2444482] |
| 23 | Cdkn2d | 1.9635 | 0.02072896 | cyclin-dependent kinase inhibitor 2D (p19, inhibits CDK4) [Source:MarkerSymbol;Acc:MGI:105387] |
| 24 | Gpr135 | 1.6098 | 0.02150685 | G protein-coupled receptor 135 [Source:MarkerSymbol;Acc:MGI:2676315] |
| 25 | Scoc | 1.8670 | 0.02150685 | short coiled-coil protein [Source:MarkerSymbol;Acc:MGI:1927654] |
| 26 | Gabrb3 | 1.3919 | 0.02375195 | gamma-aminobutyric acid (GABA-A) receptor, subunit beta 3 [Source:MarkerSymbol;Acc:MGI:95621] |
| 27 | Npm3 | -1.4788 | 0.02375195 | nucleoplasmin 3 [Source:MarkerSymbol;Acc:MGI:894653] |
| 28 | Abi3 | -1.4952 | 0.02375195 | ABI gene family, member 3 [Source:MarkerSymbol;Acc:MGI:1913860] |
| 29 | Cntnap2 | 3.1390 | 0.02375195 | contactin associated protein-like 2 [Source:MarkerSymbol;Acc:MGI:1914047] |
| 30 | Cst7 | 2.1916 | 0.02375195 | cystatin F (leukocystatin) [Source:MarkerSymbol;Acc:MGI:1298217] |
| 31 | Agtr1a | -2.0356 | 0.02375195 | angiotensin II receptor, type 1a [Source:MarkerSymbol;Acc:MGI:87964] |
| 32 | Galnt11 | 1.3890 | 0.02375195 | UDP-N-acetyl-alpha-D-galactosamine:polypeptide N-acetylgalactosaminyltransferase 11 [Source:MarkerSymbol;Acc:MGI:2444392] |
| 33 | Timp2 | 2.8844 | 0.02424609 | tissue inhibitor of metalloproteinase 2 [Source:MarkerSymbol;Acc:MGI:98753] |
| 34 | Sec14l5 | 1.6982 | 0.02551607 | - |
| 35 | LOC671569 | 2.5746 | 0.02631376 | similar to vomeronasal 2, receptor, 4 (LOC638128), mRNA [Source:RefSeq_dna;Acc:XR_004419] |
| 36 | Fbln1 | 2.3098 | 0.02859772 | fibulin 1 [Source:MarkerSymbol;Acc:MGI:95487] |
| 37 | Ccl4 | -1.9811 | 0.02921447 | chemokine (C-C motif) ligand 4 [Source:MarkerSymbol;Acc:MGI:98261] |
| 38 | Scg3 | 1.2395 | 0.03089653 | secretogranin III [Source:MarkerSymbol;Acc:MGI:103032] |
| 39 | Ccdc100 | 2.3255 | 0.03089653 | coiled-coil domain containing 100 [Source:MarkerSymbol;Acc:MGI:2147298] |
| 40 | A730008H23Rik | 2.3568 | 0.03124495 | RIKEN cDNA A730008H23 gene [Source:MarkerSymbol;Acc:MGI:3612871] |
| 41 | Mamdc4 | 1.7744 | 0.03280693 | - |
| 42 | C78409 | 1.2910 | 0.03362452 | expressed sequence C78409 [Source:MarkerSymbol;Acc:MGI:2143920] |
| 43 | Mttp | -1.1711 | 0.03388515 | microsomal triglyceride transfer protein [Source:MarkerSymbol;Acc:MGI:106926] |
| 44 | 9530080O11Rik | 1.5041 | 0.0351938 | RIKEN cDNA 9530080O11 gene [Source:MarkerSymbol;Acc:MGI:2441751] |
| 45 | Neud4 | -1.4655 | 0.03570964 | neuronal d4 domain family member [Source:MarkerSymbol;Acc:MGI:1352748] |
| 46 | Maged1 | 1.5833 | 0.03570964 | melanoma antigen, family D, 1 [Source:MarkerSymbol;Acc:MGI:1930187] |
| 47 | Cyc1 | -1.5547 | 0.0361207 | cytochrome c-1 [Source:MarkerSymbol;Acc:MGI:1913695] |
| 48 | Sparcl1 | 1.8777 | 0.0361207 | SPARC-like 1 (mast9, hevin) [Source:MarkerSymbol;Acc:MGI:108110] |
| 49 | 2310005P05Rik | 2.8277 | 0.036475 | RIKEN cDNA 2310005P05 gene [Source:MarkerSymbol;Acc:MGI:1914734] |
| 50 | Olfr129 | 1.7202 | 0.03712421 | olfactory receptor 129 [Source:MarkerSymbol;Acc:MGI:2177512] |
| 51 | Olfr786 | -2.4052 | 0.03714059 | olfactory receptor 786 [Source:MarkerSymbol;Acc:MGI:3030620] |
| 52 | Sct | 1.9484 | 0.037919 | secretin [Source:MarkerSymbol;Acc:MGI:99466] |
| 53 | Rbm12b | 1.2897 | 0.03879637 | RNA binding motif protein 12B [Source:MarkerSymbol;Acc:MGI:1919647] |
| 54 | Osbpl9 | -1.0523 | 0.03884234 | oxysterol binding protein-like 9 [Source:MarkerSymbol;Acc:MGI:1923784] |
| 55 | C3 | 2.1774 | 0.03925519 | complement component 3 [Source:MarkerSymbol;Acc:MGI:88227] |
| 56 | Fkbp14 | -1.2013 | 0.03925519 | FK506 binding protein 14 [Source:MarkerSymbol;Acc:MGI:2387639] |
| 57 | Q32P07_MOUSE | -1.6396 | 0.04035584 | similar to reduced expression 2 (LOC673430), mRNA [Source:RefSeq_dna;Acc:XR_004229] |
| 58 | Nab2 | 1.0093 | 0.04035584 | Ngfi-A binding protein 2 [Source:MarkerSymbol;Acc:MGI:107563] |
| 59 | Rsc1a1 | 1.4573 | 0.0404147 | regulatory solute carrier protein, family 1, member 1 [Source:MarkerSymbol;Acc:MGI:3526447] |
| 60 | Il17f | 1.6851 | 0.04063475 | interleukin 17F [Source:MarkerSymbol;Acc:MGI:2676631] |
| 61 | Grpel2 | 2.0741 | 0.04063475 | GrpE-like 2, mitochondrial [Source:MarkerSymbol;Acc:MGI:1334416] |
| 62 | Rps17 | -1.2378 | 0.04063475 | ribosomal protein S17 [Source:MarkerSymbol;Acc:MGI:1309526] |
| 63 | Sypl2 | 1.2807 | 0.04063475 | synaptophysin-like 2 [Source:MarkerSymbol;Acc:MGI:1328311] |
| 64 | Gpr133 | 1.7154 | 0.04063475 | G protein-coupled receptor 133 [Source:MarkerSymbol;Acc:MGI:3041203] |
| 65 | Dusp3 | -2.0175 | 0.04063475 | dual specificity phosphatase 3 (vaccinia virus phosphatase VH1-related) [Source:MarkerSymbol;Acc:MGI:1919599] |
| 66 | 4933403G14Rik | -1.6077 | 0.04063475 | RIKEN cDNA 4933403G14 gene [Source:MarkerSymbol;Acc:MGI:1921643] |
| 67 | Chrac1 | -1.2278 | 0.04063475 | chromatin accessibility complex 1 [Source:MarkerSymbol;Acc:MGI:2135796] |
| 68 | Asb17 | -1.9685 | 0.04174006 | ankyrin repeat and SOCS box-containing protein 17 [Source:MarkerSymbol;Acc:MGI:1914022] |
| 69 | Nhlh1 | -2.5056 | 0.04203214 | nescient helix loop helix 1 [Source:MarkerSymbol;Acc:MGI:98481] |
| 70 | D17H6S56E-3 | -1.5934 | 0.04203214 | Protein G7c precursor. [Source:Uniprot/SWISSPROT;Acc:Q9JHA8] |
| 71 | Hnrph1 | 2.3122 | 0.04203214 | heterogeneous nuclear ribonucleoprotein H1 [Source:MarkerSymbol;Acc:MGI:1891925] |
| 72 | 5730419I09Rik | -2.1172 | 0.04203214 | RIKEN cDNA 5730419I09 gene [Source:MarkerSymbol;Acc:MGI:1921991] |
| 73 | 2310016F22Rik | -1.6474 | 0.04203214 | RIKEN cDNA 2310016F22 gene [Source:MarkerSymbol;Acc:MGI:1919148] |
| 74 | Pcyt1b | -1.5782 | 0.04203214 | phosphate cytidylyltransferase 1, choline, beta isoform [Source:MarkerSymbol;Acc:MGI:2147987] |
| 75 | Cant1 | 1.8062 | 0.04203214 | calcium activated nucleotidase 1 [Source:MarkerSymbol;Acc:MGI:1923275] |
| 76 | Rad9b | -1.2536 | 0.04203214 | RAD9 homolog B (S. cerevisiae) [Source:MarkerSymbol;Acc:MGI:2385231] |
| 77 | 4921524J17Rik | -1.5437 | 0.04203214 | RIKEN cDNA 4921524J17 gene [Source:MarkerSymbol;Acc:MGI:1913964] |
| 78 | H13 | -1.6934 | 0.04203214 | histocompatibility 13 [Source:MarkerSymbol;Acc:MGI:95886] |
| 79 | Clca1 | -1.7077 | 0.04284047 | chloride channel calcium activated 1 [Source:MarkerSymbol;Acc:MGI:1316732] |
| 80 | - | -1.9707 | 0.04337675 | similar to Elongation factor 2 (EF-2) (LOC435640), mRNA [Source:RefSeq_dna;Acc:XR_003342] |
| 81 | Olfr1243 | -1.6393 | 0.04438534 | olfactory receptor 1243 [Source:MarkerSymbol;Acc:MGI:3031077] |
| 82 | V2r1b | -1.6910 | 0.04537002 | vomeronasal 2, receptor, 1b [Source:MarkerSymbol;Acc:MGI:2678394] |
| 83 | Olfr1507 | -1.6109 | 0.04544008 | olfactory receptor 1507 [Source:MarkerSymbol;Acc:MGI:3031341] |
| 84 | Guf1 | 1.6631 | 0.04544008 | GUF1 GTPase homolog (S. cerevisiae) [Source:MarkerSymbol;Acc:MGI:2140726] |
| 85 | IGKV19-93 | -2.1451 | 0.04544008 | Immunoglobulin Kappa light chain V gene segment [Source:IMGT/GENE-DB;Acc:IGKV19-93] |
| 86 | Sprn | 1.7067 | 0.04544008 | shadow of prion protein [Source:MarkerSymbol;Acc:MGI:3582583] |
| 87 | Psmd2 | -1.3837 | 0.04544008 | proteasome (prosome, macropain) 26S subunit, non-ATPase, 2 [Source:MarkerSymbol;Acc:MGI:1096584] |
| 88 | Setd7 | 1.5956 | 0.04544008 | SET domain containing (lysine methyltransferase) 7 [Source:MarkerSymbol;Acc:MGI:1920501] |
| 89 | 5730593N15Rik | 2.8038 | 0.04544008 | RIKEN cDNA 5730593N15 gene [Source:MarkerSymbol;Acc:MGI:1924833] |
| 90 | Mettl2 | 1.3873 | 0.04585038 | methyltransferase like 2 [Source:MarkerSymbol;Acc:MGI:1289171] |
| 91 | Alx4 | -1.4620 | 0.04585038 | aristaless 4 [Source:MarkerSymbol;Acc:MGI:108359] |
| 92 | OTTMUSG00000005421 | -1.8677 | 0.04656746 | predicted gene, OTTMUSG00000005421 [Source:MarkerSymbol;Acc:MGI:3651509] |
| 93 | V1rh14 | -2.0174 | 0.04656746 | vomeronasal 1 receptor, H14 [Source:MarkerSymbol;Acc:MGI:2159677] |
| 94 | Abca8a | 2.4172 | 0.04664016 | ATP-binding cassette, sub-family A (ABC1), member 8a [Source:MarkerSymbol;Acc:MGI:2386846] |
| 95 | Lcmt1 | 1.2574 | 0.04664016 | leucine carboxyl methyltransferase 1 [Source:MarkerSymbol;Acc:MGI:1353593] |
| 96 | Ckap2l | -1.0778 | 0.04664016 | cytoskeleton associated protein 2-like [Source:MarkerSymbol;Acc:MGI:1917716] |
| 97 | Ccdc88 | 1.1314 | 0.04664016 | coiled-coil domain containing 88 [Source:MarkerSymbol;Acc:MGI:1925567] |
| 98 | Plekhh3 | -1.4464 | 0.04664016 | pleckstrin homology domain containing, family H (with MyTH4 domain) member 3 [Source:MarkerSymbol;Acc:MGI:2384950] |
| 99 | Ywhaz | -1.6049 | 0.04664016 | tyrosine 3-monooxygenase/tryptophan 5-monooxygenase activation protein, zeta polypeptide [Source:MarkerSymbol;Acc:MGI:109484] |
| 100 | Slc9a3r2 | 1.4975 | 0.04664016 | solute carrier family 9 (sodium/hydrogen exchanger), isoform 3 regulator 2 [Source:MarkerSymbol;Acc:MGI:1890662] |
| 101 | Nasp | -1.5761 | 0.04703646 | nuclear autoantigenic sperm protein (histone-binding) [Source:MarkerSymbol;Acc:MGI:1355328] |
| 102 | 2610301G19Rik | 1.6169 | 0.04703646 | RIKEN cDNA 2610301G19 gene [Source:MarkerSymbol;Acc:MGI:2444228] |
| 103 | Fbxl4 | -1.6864 | 0.04703646 | F-box and leucine-rich repeat protein 4 [Source:MarkerSymbol;Acc:MGI:2140367] |
| 104 | D8Ertd457e | 1.1965 | 0.04757445 | Zinc finger protein KIAA1802. [Source:Uniprot/SWISSPROT;Acc:Q8K327] |
| 105 | 4933439C20Rik | -1.8217 | 0.04757445 | RIKEN cDNA 4933439C20 gene [Source:MarkerSymbol;Acc:MGI:1914026] |
| 106 | ENSMUSG00000056739 | -1.9608 | 0.04780879 | predicted gene, ENSMUSG00000056739 [Source:MarkerSymbol;Acc:MGI:3642351] |
| 107 | Jmy | 1.1837 | 0.04920527 | junction-mediating and regulatory protein [Source:MarkerSymbol;Acc:MGI:1913096] |
