# Supplementary material for: Molecular Profiling of Single Sca-1+/CD34+,− Cells—The Putative Murine Lung Stem Cells
Source: PLoS One. 2013 Dec 31;8(12):e83917. doi: 10.1371/journal.pone.0083917 (PMC3877111; doi:10.1371/journal.pone.0083917)
Supplement: Table S3 — Differentially expressed genes (Sca1+/CD34+ vs Sca1+/CD34–). (DOC) [file pone.0083917.s004.doc]

**Table S3: Differentially expressed genes (Sca1+/CD34+ vs Sca1+/CD34-)**

|  | **Gene symbol** | **Fold change** | **Adj. P-value** | **Gene description** |
| --- | --- | --- | --- | --- |
| 1 | Esd | 2.8242 | 0.01899187 | esterase D/formylglutathione hydrolase [Source:MarkerSymbol;Acc:MGI:95421] |
| 2 | Kbtbd10 | 4.0676 | 0.03330934 | kelch repeat and BTB (POZ) domain containing 10 [Source:MarkerSymbol;Acc:MGI:2683854] |
| 3 | NP_001032841.1 | 2.6280 | 0.04392966 | beta-defensin 27 [Source:RefSeq_peptide;Acc:NP_001032841] |
| 4 | Slc8a3 | 2.4816 | 0.04745900 | solute carrier family 8 (sodium/calcium exchanger), member 3 [Source:MarkerSymbol;Acc:MGI:107976] |
| 5 | Spast | 3.3151 | 0.04813769 | spastin [Source:MarkerSymbol;Acc:MGI:1858896] |
| 6 | 1110007L15Rik | 1.8483 | 0.04813769 | RIKEN cDNA 1110007L15 gene [Source:MarkerSymbol;Acc:MGI:1914854] |
| 7 | Exoc6b | 1.7033 | 0.04813769 | exocyst complex component 6B [Source:MarkerSymbol;Acc:MGI:1923164] |
| 8 | Scoc | 2.2671 | 0.04813769 | short coiled-coil protein [Source:MarkerSymbol;Acc:MGI:1927654] |
