# Supplementary material for: Molecular Profiling of Single Sca-1+/CD34+,− Cells—The Putative Murine Lung Stem Cells
Source: PLoS One. 2013 Dec 31;8(12):e83917. doi: 10.1371/journal.pone.0083917 (PMC3877111; doi:10.1371/journal.pone.0083917)
Supplement: Table S4 — Detected mRNA transcripts in isolated single cells. (DOC) [file pone.0083917.s005.doc]

**Table S4: Detected mRNA transcripts in isolated single cells**

|  |  |  | **IF Staining** | | **Marker PCR** | | | | | | | | |
| --- | --- | --- | --- | --- | --- | --- | --- | --- | --- | --- | --- | --- | --- |
| **cell ID** | **cell type** | **microarray batch** | **Sca-1** | **CD34** | ***Sca-1*** | ***CD34*** | ***CD31*** | ***CD45*** | ***Epcam*** | ***CD90*** | ***Pdgfrα*** | ***Itga6*** | ***Sftpc*** |
| 1 | *Sca1-/CD34+* |  | 1 | 0 | 0 | 1 | 0 | 0 | 1 | 0 | 1 | 0 | 0 |
| 2 | *Sca1-/CD34+* |  | 1 | 0 | 0 | 1 | 0 | 0 | 0 | 0 | 1 | 0 | 0 |
| 3 | *Sca1-/CD34+* |  | 1 | 0 | 0 | 1 | 0 | 0 | 0 | 1 | 0 | 0 | 0 |
| 4 | *Sca1-/CD34+* |  | 0 | 1 | 0 | 1 | 0 | 0 | 0 | 0 | 1 | 0 | 1 |
| 5 | *Sca1-/CD34+* |  | 1 | 0 | 0 | 1 | 0 | 0 | 0 | 0 | 0 | 0 | 0 |
| 6 | *Sca1-/CD34+* |  | 0 | 1 | 0 | 1 | 0 | 0 | 0 | 0 | 0 | 0 | 0 |
| 7 | *Sca1-/CD34+* |  | 1 | 0 | 0 | 1 | 0 | 0 | 0 | 0 | 0 | 0 | 0 |
| 8 | *Sca1+/CD34+* | 20207 | 1 | 0 | 1 | 1 | 0 | 0 | 0 | 0 | 0 | 0 | 0 |
| 9 | *Sca1+/CD34+* | 20205/20207 | 0 | 1 | 1 | 1 | 0 | 0 | 0 | 0 | 1 | 0 | 1 |
| 10 | *Sca1+/CD34+* | 20205 | 0 | 1 | 1 | 1 | 0 | 0 | 0 | 0 | 1 | 1 | 1 |
| 11 | *Sca1+/CD34+* | n.d. | 0 | 1 | 1 | 1 | 0 | 0 | 0 | 1 | 0 | 0 | 1 |
| 12 | *Sca1+/CD34+* | n.d. | 0 | 1 | 1 | 1 | 0 | 0 | 0 | 0 | 1 | 0 | 1 |
| 13 | *Sca1+/CD34+* | 20205 | 0 | 1 | 1 | 1 | 0 | 0 | 0 | 0 | 0 | 0 | 0 |
| 14 | *Sca1+/CD34+* | 20339 | 0 | 1 | 1 | 1 | 0 | 0 | 0 | 0 | 0 | 0 | 1 |
| 15 | *Sca1+/CD34+* | 20339 | 0 | 1 | 1 | 1 | 0 | 0 | 0 | 0 | 0 | 0 | 0 |
| 16 | *Sca1+/CD34+* | 20205 | 0 | 1 | 1 | 1 | 0 | 0 | 0 | 0 | 1 | 0 | 1 |
| 17 | *Sca1+/CD34+* | 20339 | 0 | 1 | 1 | 1 | 0 | 0 | 0 | 0 | 1 | 0 | 1 |
| 18 | *Sca1+/CD34+* |  | 1 | 0 | 1 | 1 | 0 | 0 | 0 | 0 | 1 | 0 | 0 |
| 19 | *Sca1+/CD34+* |  | 1 | 0 | 1 | 1 | 0 | 0 | 0 | 0 | 1 | 0 | 0 |
| 20 | *Sca1+/CD34+* |  | 0 | 1 | 1 | 1 | 0 | 0 | 0 | 0 | 0 | 0 | 0 |
| 21 | *Sca1+/CD34+* |  | 0 | 1 | 1 | 1 | 0 | 0 | 0 | 0 | 0 | 0 | 1 |
| 22 | *Sca1+/CD34+* |  | 0 | 1 | 1 | 1 | 0 | 0 | 0 | 0 | 0 | 0 | 1 |
| 23 | *Sca1+/CD34+* |  | 1 | 0 | 1 | 1 | 0 | 0 | 0 | 0 | 1 | 0 | 1 |
| 24 | *Sca1+/CD34+* |  | 0 | 1 | 1 | 1 | 0 | 0 | 0 | 0 | 1 | 0 | 1 |
| 25 | *Sca1+/CD34-* | n.d. | 0 | 1 | 1 | 0 | 0 | 0 | 0 | 0 | 0 | 0 | 0 |
| 26 | *Sca1+/CD34-* | n.d. | 0 | 1 | 1 | 0 | 0 | 0 | 0 | 1 | 0 | 0 | 0 |
| 27 | *Sca1+/CD34-* | n.d. | 1 | 0 | 1 | 0 | 0 | 0 | 1 | 0 | 0 | 0 | 0 |
| 28 | *Sca1+/CD34-* | n.d. | 0 | 1 | 1 | 0 | 0 | 0 | 0 | 0 | 0 | 0 | 0 |
| 29 | *Sca1+/CD34-* | 20331 | 1 | 0 | 1 | 0 | 0 | 0 | 0 | 0 | 0 | 0 | 1 |
| 30 | *Sca1+/CD34-* | 20331 | 0 | 1 | 1 | 0 | 0 | 0 | 1 | 0 | 0 | 0 | 1 |
| 31 | *Sca1+/CD34-* | 20331 | 1 | 0 | 1 | 0 | 0 | 0 | 0 | 0 | 0 | 0 | 1 |
| 32 | *Sca1+/CD34-* |  | 1 | 0 | 1 | 0 | 0 | 0 | 0 | 0 | 0 | 0 | 1 |
| 33 | *Sca1+/CD34-* |  | 1 | 0 | 1 | 0 | 0 | 0 | 0 | 0 | 0 | 0 | 0 |
| 34 | *Sca1+/CD34-* |  | 0 | 1 | 1 | 0 | 0 | 0 | 0 | 0 | 0 | 0 | 1 |
| 35 | *Sca1+/CD34-* |  | 1 | 0 | 1 | 0 | 0 | 0 | 0 | 0 | 1 | 0 | 1 |
| 36 | *Sca1+/CD34-* |  | 1 | 0 | 1 | 0 | 0 | 0 | 1 | 0 | 0 | 0 | 0 |
| 37 | *Sca1+/CD34-* |  | 1 | 0 | 1 | 0 | 0 | 0 | 1 | 0 | 0 | 0 | 0 |
| 38 | *Sca1+/CD34-* |  | 1 | 0 | 1 | 0 | 0 | 0 | 1 | 0 | 0 | 0 | 0 |
| 39 | *Sca1+/CD34-* |  | 1 | 0 | 1 | 0 | 0 | 0 | 0 | 0 | 0 | 0 | 0 |
| 40 | *Sca1+/CD34-* |  | 1 | 0 | 1 | 0 | 0 | 0 | 0 | 0 | 0 | 0 | 1 |
| 41 | *Sca1+/CD34-* |  | 0 | 1 | 1 | 0 | 0 | 0 | 0 | 0 | 1 | 0 | 0 |
| 42 | *Sca1+/CD34-* |  | 1 | 0 | 1 | 0 | 0 | 0 | 0 | 0 | 0 | 0 | 0 |
| 43 | *Sca1+/CD34-* |  | 1 | 0 | 1 | 0 | 0 | 0 | 0 | 1 | 0 | 0 | 0 |
| 44 | *Sca1+/CD34-* |  | 1 | 0 | 1 | 0 | 0 | 0 | 1 | 0 | 0 | 0 | 0 |
| 45 | *Sca1+/CD34-* |  | 0 | 1 | 1 | 0 | 0 | 0 | 0 | 0 | 1 | 1 | 0 |
| 46 | *Sca1+/CD34-* |  | 0 | 1 | 1 | 0 | 0 | 0 | 0 | 0 | 1 | 0 | 0 |
| 47 | *Sca1-/CD34-* | 20205/20207 |  |  | 0 | 0 | 0 | 0 | 0 | 0 | 0 | 0 | 1 |
| 48 | *Sca1-/CD34-* | 20205/20207 |  |  | 0 | 0 | 0 | 0 | 0 | 0 | 0 | 0 | 1 |
| 49 | *Sca1-/CD34-* | n.d. |  |  | 0 | 0 | 0 | 0 | 0 | 0 | 0 | 0 | 0 |
| 50 | *Sca1-/CD34-* | 20205/20207 |  |  | 0 | 0 | 0 | 0 | 1 | 0 | 1 | 0 | 1 |
| 51 | *Sca1-/CD34-* | n.d. |  |  | 0 | 0 | 0 | 0 | 0 | 0 | 0 | 0 | 0 |
| 52 | *Sca1-/CD34-* | 20205/20207 |  |  | 0 | 0 | 0 | 0 | 0 | 0 | 1 | 0 | 1 |
| 53 | *Sca1-/CD34-* | n.d. |  |  | 0 | 0 | 0 | 0 | 1 | 0 | 0 | 1 | 1 |
| 54 | *Sca1-/CD34-* | 20331 |  |  | 0 | 0 | 0 | 0 | 0 | 0 | 0 | 0 | 0 |
| 55 | *Sca1-/CD34-* | 20331 |  |  | 0 | 0 | 0 | 0 | 1 | 0 | 0 | 0 | 1 |
| 56 | *Sca1-/CD34-* | 20331 |  |  | 0 | 0 | 0 | 0 | 1 | 0 | 0 | 0 | 1 |
| 57 | *Sca1-/CD34-* | 20331 |  |  | 0 | 0 | 0 | 0 | 0 | 0 | 0 | 0 | 0 |
| 58 | *Sca1-/CD34-* | 20331 |  |  | 0 | 0 | 0 | 0 | 0 | 0 | 0 | 0 | 1 |
| 59 | *Sca1-/CD34-* |  |  |  | 0 | 0 | 0 | 0 | 0 | 0 | 0 | 0 | 0 |
| 60 | *Sca1-/CD34-* |  |  |  | 0 | 0 | 0 | 0 | 0 | 0 | 0 | 0 | 0 |
| 61 | *Sca1-/CD34-* |  |  |  | 0 | 0 | 0 | 0 | 0 | 0 | 0 | 0 | 0 |
| 62 | *Sca1-/CD34-* |  |  |  | 0 | 0 | 0 | 0 | 0 | 0 | 0 | 0 | 1 |
| 63 | *Sca1-/CD34-* |  |  |  | 0 | 0 | 0 | 0 | 0 | 0 | 0 | 0 | 0 |
| 64 | *Sca1-/CD34-* |  |  |  | 0 | 0 | 0 | 0 | 0 | 0 | 0 | 0 | 1 |
| 65 | *Sca1-/CD34-* |  |  |  | 0 | 0 | 0 | 0 | 0 | 1 | 0 | 0 | 0 |
| 66 | *Sca1-/CD34-* |  |  |  | 0 | 0 | 0 | 0 | 0 | 0 | 0 | 0 | 0 |
| 67 | *Sca1-/CD34-* |  |  |  | 0 | 0 | 0 | 0 | 0 | 0 | 0 | 0 | 0 |
